# Extracellular Enzyme of Endophytic Fungi Isolated from *Ziziphus spina* Leaves as Medicinal Plant

**DOI:** 10.1155/2022/2135927

**Published:** 2022-07-06

**Authors:** Sumaiya Naeema Hawar

**Affiliations:** Biology Department, College of Education for Pure Science/Ibn Al- Haitham, University of Baghdad, Baghdad, Iraq

## Abstract

Endophytic fungi live inside plants or any part of them without creating any visible pathogenic signs. Endophytic fungi are found within medicinal plants and have shown strong biologic activity, such as anticancer and antioxidant activities, as well as producing extracellular enzymes. In this study, different fungal strains were isolated from the leaves of the medicinal plant Ziziphus spina, including *Aspergillus flavus*, *Aspergillus fumigatus*, *Aspergillus niger*, *Cladosporium* sp., *Rhizopus* sp., and *Mucor* sp. Extracellular enzymes have been quantified using agar plate-based methods in which fungi were grown in specified growth media to detect the enzymes produced. The results showed that *A. niger* has the highest ability to produce amylase, *Cladosporium* sp. has the highest ability to produce protease and pectinase, *Rhizopus* and *Mucor* sp. have the highest ability to produce cellulase, and *A. niger* and *Cladosporium* sp. have the same ability to produce lipase and laccase. The ability of medicinal plant endophytic fungi to produce extracellular enzymes has great therapeutic potential in clinical microbiology. Some of the isolates showed great activity in secreting particular enzymes, indicating that the enzymes of these fungi could be used in a variety of applications.

## 1. Introduction

Endophytes are all microorganisms that invade the internal tissues of living plants without causing any immediate disease symptoms or negative effects on their host; this definition is the most comprehensive and most widely used [1]. Endophytic fungi contribute to the protection of their hosts against pathogenic microorganisms by competing for their habitat and food sources [2]. Furthermore, it works to prevent infection by pathogenic organisms through the rapid invasion of its hosts and the depletion of available nutrients that are necessary for the growth of pathogenic microorganisms [3]. The endophytic fungi can contribute to the protection of their hosts against insects and animals [4, 5], this is through the production of some toxic compounds that render the host inedible to insects and animals [6]. Several studies indicate that the extracellular enzymes amylase, pectinase, cellulases, laccase, and protease are produced by endophytic fungi as a strategy for resistance to infections and getting sustenance from the host [7, 8]. The bioremediation processes that depend on the enzyme systems of microorganisms, including endophytic fungi, have received great attention, which indicates that these fungi can be used for detoxification or in the bioremediation of industrial and agricultural waste and other polluting compounds [8].

Fungi have the ability to produce industrial enzymes, antimicrobial agents, and microbial biomass and have evolved the ability to synthesize enzymes and natural products as novel metabolites. Fungal enzymes such as amylases, lipases, and cellulases can act directly as antimicrobials, or they can create intermediate molecules with antimicrobial activity [9]. Moreover, fungal enzymes are used in food production to preserve food and shelf life without affecting the nutritional content [10]. The production of enzymes from endophytic fungus for commercial use is an unexplored field that also represents one of the most recent sources of enzymes with various activities. Fungi are a major source of enzymes used in the food and beverage sector, as well as leather, paper, textiles, and other industries [11].

Medicinal plants are considered pharmaceutical agents, so it is important for the development of drugs. According to the WHO, herbal medicine and traditional medicine are utilized by around 80% of the world's population in developing nations for primary healthcare [12]. *Ziziphus spina* is used to treat fractures, chest pain, headaches, blisters, and bruises [13]. The genus *Ziziphus* belongs to the Rhamnaceae family, with *Z. spina-christi*, the thorns of which are said to have been used to make the crown for Holy Christ. These species are generally spiny shrubs but sometimes form small trees that strongly resist heat and drought. These are found in the tropical regions of Asia and Africa, either grown in patches or wild [14, 15]. The genus *Ziziphus* is known for its medicinal properties as an antimicrobial, hypertensive, antioxidant, hypoglycemic, antitumor, antiinflammatory, and an immune system stimulant and liver protective agent [16]. The crushed leaves of *Ziziphus* are boiled in water and given to women with a retained placenta for their oxytocic properties or prolonged labor [17]. Iron (7.2 mg 100/g dry weight), calcium (1270 mg 100/g dry weight), and magnesium (169 mg 100/g) are all abundant in the leaves [18]. This plant has recently received a lot of attention because of its nutritional value and ancient medicinal purposes, so the current study aimed to evaluate the efficiency of endophytic fungi isolated from the *Ziziphus spina* leaves plant (as a medicinal plant) in the production of many extracellular enzymes and may be considered a new source of enzymes that are used in industrial and pharmaceutical applications.

## 2. Material and Methods

All chemicals, materials, media, and solvents used in this study were obtained from Sigma-Aldrich (UK).

### 2.1. Collection of Plant Samples

Healthy fresh and green leaves of *Ziziphus spina* in old age were obtained from the botanical garden for postgraduate studies at the College of Education for Pure Sciences Ibn Al-Haitham/University of Baghdad. The leaves were free from any pathogenic infection and picked from an area free from pollution from June 1 until August 30 (2021). The selected plants were identified at the Department of Biological College of Education for pure science Ibn-Al-Haitham/University of Baghdad.

### 2.2. Isolation of Endophyte Fungi from *Ziziphus spina* Leaves

After collecting, the leaves were cleaned thoroughly with tap water, then surface-disinfected with 70% ethanol for 1 minute and sodium hypochlorite solution (2%) for 5 minutes, and finally washed twice with sterilized distilled water for 3 minutes. The leaves were dried thoroughly on sterilized filter paper, and then cut into little pieces of 10 mm in length, with four pieces of each sample cultured in three replicates on potato dextrose agar medium with chloramphenicol (0.05 mg/ml). Under monitoring, the plates were incubated at 28°C for 7–10 days until mycelia developed around the plant tissues. The fungal growth on plant tissues was subsequently inoculated on PDA for the formation of pure colonies as well as further identification and preservation [19].

### 2.3. Identification of Endophytic Fungi

The isolated fungal genera were identified based on morphological characteristics including colony morphology, mycelia, colony color, and texture according to the taxonomy guide and microscopic characteristics using light microscope observations of mycelia and asexual/sexual spores. The identification of fungal genera was done with the diagnostic keys [20–25].

### 2.4. Determination of the Production of Extracellular Enzymes

The experimental design was completely randomized in a factorial scheme (media, isolates, and enzymes), with three replications, each consisting of a plastic Petri dish containing the medium and the fungus isolate. Control treatments were all the media not colonized by the fungi strain. Extracellular enzymes have been qualitatively measured through agar plate-based methods in which fungi have grown in specific growth media to detect the enzymes produced. The functional role of exogenous enzymes by cultured fungi was measured by growing the fungi on a PDA medium for 7 days and then culturing a 5 mm disc on solid culture media containing substrate materials (soluble starch, olive oil, gelatin, CMC, 1-napthol) for 5–7 days. The region of enzyme activity that surrounds the fungal colony. The specific media reported in the study to produce and detect enzymes in the medium are described as follows.

#### 2.4.1. Amylase

The activity of amylase was measured by growing the studied fungi on glucose yeast extract peptone agar (GYP) with 0.2% soluble starch. After the incubation period, the dishes were flooded with reagent material consisting of iodine (1%) and potassium iodide (2%). The transparent circle surrounding the colony indicates the production of amylase [11].

#### 2.4.2. Lipase

To measure the activity of lipase, the fungi were grown on peptone agar medium supplemented with olive oil (1%), which was sterilized separately and then added to the medium. After incubation, a sedimentation ring is observed surrounding the fungal colony as a result of the precipitation of calcium salts, which indicates the activity of lipase [26].

#### 2.4.3. Protease

The activity of protease was measured by growing the fungi on a GYP medium supplemented with gelatin (8%), which was sterilized alone and then added to the GYP medium; then the dishes were immersed in a saturated aqueous solution of ammonium sulfate, where a transparent circle appears around the fungal colony [26].

#### 2.4.4. Cellulase

Carboxymethyl cellulose (CMC) (0.5 g) agar medium was used to measure cellulose activity. After 5 days of incubation, the dishes were immersed in Congo red (0.2%) for 30 minutes, then 1 M sodium chloride was added for 15 minutes, and a yellow transparent circle appeared around the colony [26].

#### 2.4.5. Laccase

The GYP medium with 0.05 g of 1-napthol was used, and after the incubation, the color of the medium surrounding the colony changed from colorless to blue, indicating the activity of lacase [11].

#### 2.4.6. Pectinase

Fungi were grown on pectin agar medium. After the incubation period, the dishes were immersed in 1 aqueous solution of hexadecyl trimethylammonium bromide. The appearance of a transparent circle around the fungal colony indicates the activity of pectinase [11].

### 2.5. Evaluation Criteria

#### 2.5.1. Criteria 1

The following equation was used to determine this criterion:

(1) $$\begin{array}{c} \frac{\text{Percentage of }\deg\text{ radation }\left(\text{\%}\right)=\text{fungal colony diameter }\left(\text{mm}\right)}{\text{fungal colony diameter}+\text{degradation area}}. \end{array}$$

If the result is 1, it indicates that isolates cannot generate the enzyme. If the value was between 1 and 0.69, it means the isolates had a low level of production for this enzyme. It is well produced if the value is less than 0.69 and larger than 0.3, and it is very high if the number is less than 0.3 [27].

#### 2.5.2. Criteria 2

Using a note scale [28], the diameter of the halo formed around the fungal colonies was measured to determine enzyme activity; observe the scale. 0 indicates no production, 1 indicates a halo diameter of 1–5 mm, 2 indicates a diameter of 5–10 mm, 3 indicates a diameter of 10–20 mm, 4 indicates a diameter of 20–30 mm, and 5 indicates a diameter of greater than 30 mm. ^+++^ high, ^++^ moderate, ^+^ weak, —absent are the symbols used to estimate their production.

### 2.6. Statistical Analysis

The collecting data were analyzed using the SPSS software version 24 program; chi-square and one way ANOVA tests and (LSD) were used to find the significant differences as appropriate between the averages of data [29].

## 3. Results and Discussion

The results of the current study showed that the endophytic fungi in the leaves of the *Ziziphus spina* plant were isolated from a total of 52 leaf pieces. The total number of isolates was obtained by 26 endophytic fungi with a total colonization frequency of 50%. These fungi belong to 6 species (Table 1), including *Aspergillus niger* with a colonization frequency of 13.46%, *Aspergillus fumigatous* at 11.54%, *Rhizopus* and *Mucor* with a colonization frequency of 7.69% each, *Aspergillus flavus* at a frequency of 5.77%, and *Cladosporium* sp. at a colonization frequency of 3.85%. Many studies [11, 30, 31] have investigated the presence of endophyte fungi in medicinal plants like *Potentilla fulgens*, *Osbeckia stellata*, *Osbeckia chinensis*, *Camellia caduca*, *Schima khasiana*, *Azadirachta indica*, *Citrus limon*, *Gossypium hirsutum*, *Magnolia champaca*, *Datura stramonium*, *Ppier betle*, *Phyllanthus emblica*, *Alpinia calcarata*, *Calophyllum inophyllum*, *Bixa Orellana,* and *Catharanthus roseus*, which are an important source of these fungi. It can produce compounds similar to those in its plant families and maybe a substitute for it; the authors of [32] showed that *Gentiana macrophylla* produce the same ametabolites gentiopicrin as an endophyte associated with the host; the authors of [33] showed the extract of endophytic fungus *Paecilomyces* sp. and the host plant ginseng contains the same compound falcarinol. The diversity and density of endophytic fungi are affected by the nature of the nearby vegetation. The availability of moisture as well as the age of the plant have an effective role, as the old parts of the plant contain more aggregated fungi when compared with the young parts. Additionally, air pollution harms the presence of these fungi [34]. Most of the species isolated in this study, including *Aspergillus niger* and *A. flavus*, have been isolated in other studies as endophytes fungi in other medicinal plants, including [35] in *Melia azedarach* and the two studies of [30, 36] in the medicinal plants *Calotropis procera* and *Withania somnifera*. The fungus *Cladosporium australis* was also isolated as a fungus cultured in the twigs of *Withania somnifera* and Sidr leaves [19].

As for the effectiveness of these fungi in secreting extracellular enzymes, the results revealed that these fungi varied in the secretion of enzymes in the medium in terms of types and effectiveness. Whereas 47.51% of these fungi secreted amylase, 44.75% secreted protease, 51% secreted cellulase, and 68%, 22%, and 25% secreted lipase, pectinase, and lacase, respectively (Table 2). Some of the isolates showed high activity in secreting some enzymes, like *A. niger* with activity in producing amylase and lipase. This indicates the possibility of exploiting the enzymes of these fungi in many applications after their separation and characterization.

### 3.1. Amylase Activity

**Figure 1:**
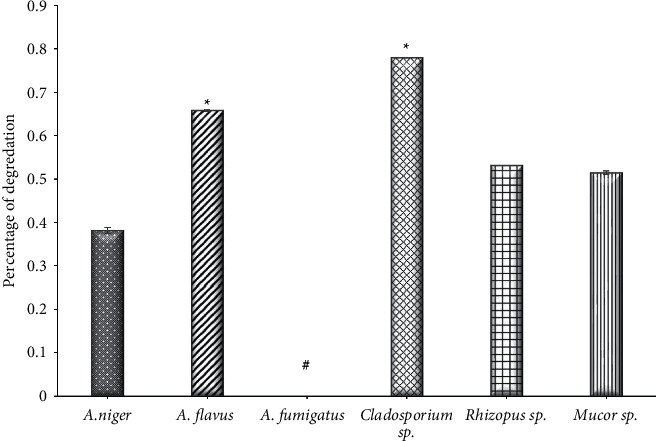
The activity of amylase of endophytic fungi isolated from *Ziziphus spina* leaves on solid medium. ^*∗*^Significant difference with (*A. niger*, (A) f*umigatus, Rhizopus sp., And Mucor sp.*), at *P* < 0.001. ^#^Significant difference with all other groups at *P* < 0.001.

The statistical analysis of the collected data of the current study revealed there are significant differences between the amylase-producing isolates (*P* < 0.001), as it was found that the most strains that produced amylase were *A. niger* with a degradation percentage of 0.3813 followed by *Mucor* sp. (0.5143) and *Rhizopus* sp. (0.5310), while the remaining strains had poor productivity; *A. fumigatus* did not show the ability to produce amylase (Figure 1). This is consistent with the results of [26] that out of 11 strains, only eight strains were positive in terms of the amylase activity isolated from the leaves of medicinal plants. Furthermore, the findings of [37] found that out of nine strains, only three were able to produce amylase enzyme from endophytic fungi that were isolated from medicinal plant leaves. Among these fungi were *Aspergillus* sp, *Rhizoctonia* sp., and *Chaetomium*; also [11] revealed that amylase activity produced by endophytic fungi helps to destroy the starch present in the host plant when it is old. Moreover, [38] showed that the most highly effective endophytic fungi for amylase were *Rhizoctonia stolonifer*, followed by *Aspergillus niger* and *Penicillium variotii*. Amylase was firstly applied medicinally in treating digestive disorders and is the world premiere in enzyme production for commercial application [39]. This enzyme is also used in food industries such as improving pastries and removing starch from sugar, as these enzymes enter the textile industry, paper and detergent industry, and preparation of animal feed.

### 3.2. Lipase activity

**Figure 2:**
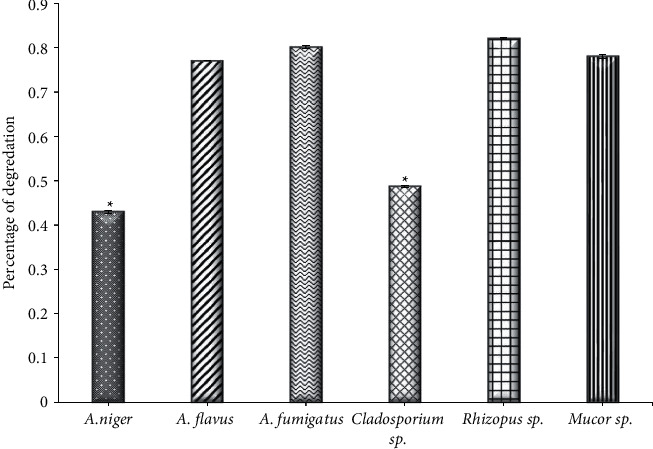
The activity of lipase of endophytic fungi isolated from *Ziziphus spina* leaves on solid medium. ^*∗*^Significant difference with (*A. flavus,* (A) *fumigatus, Rhizopus sp., and Mucor sp.*), at *P* < 0.001.

Through the results of the current study, significant differences (*P* < 0.001) between the fungal isolates that produced lipase were found; the most endophytic fungi isolated from *Ziziphus spina* leaves that produced the lipase enzyme were *A. niger* with a degradation percentage of 0.430 and *Cladosporium* sp. (0.4873), while the remaining strains had a weak production of this enzyme (Figure 2). The results of [37] found that of all nine strains isolated from several medicinal plants, only one strain, which is *Cladosporium* sp., was able to produce the lipase, while the results of [11] showed that almost half of the strains obtained from five types of medicinal plants were able to produce lipase with varying degrees of enzyme activity, as *Isaria* sp. was the most productive strain, followed by *Alternaria* sp. In contrast, the observations of [38] showed weak productivity of the fungal isolates obtained from seven types of oilseeds, and only 40 strains produced lipase enzyme, while 100% of the 5 endophytic fungal strains that were isolated from the leaves of *Croton oblangiofolius* in Thailand were able to produce lipase [40]. Lipases are implemented in vast commercial applications, including cosmetics additives and detergents, medical applications, fine chemical production, paper pitching, wastewater treatment, leather de-fating, and biodiesel production [41, 42]. The application of lipase as an ecofriendly alternative to traditional fuel in biodiesel production.

### 3.3. Protease Activity

**Figure 3:**
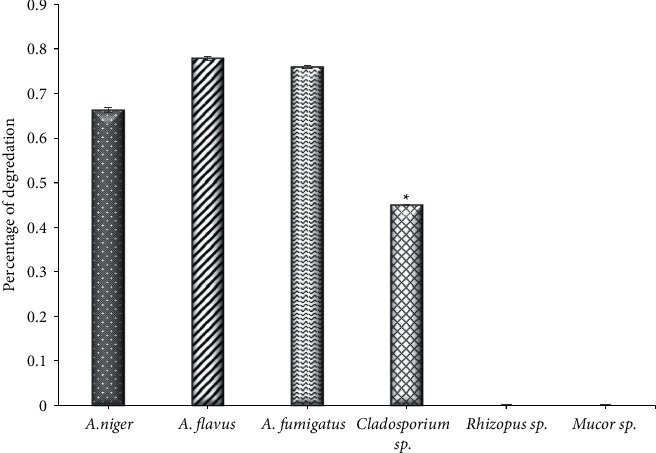
The activity of protease of endophytic fungi isolated from Ziziphus spina leaves on solid medium. ^*∗*^Significant difference with all other groups at *P* < 0.001.

The findings of this result detected a significant difference (*P* < 0.001) in the protease produced by the isolated fungi; it was found that the most protease-producing strains were *Cladosporium* sp. with a degradation percentage of 0.450, while the remaining strains appeared weak; the two strains, *Mucor* sp. and *Rhizopus* sp., did not show any ability to produce this enzyme (Figure 3). The results of [43] found that out of 20 fungal isolates, 50% of them were positive for the production of protease, and these results are similar to the results of [37] in that not all isolates are capable of producing protease, whereas some of them, such as *Rhizoctonia, Aspergillus,* and *Cladosporium,* had medium activity for this enzyme, while *Biosporus* sp. had high activity. On the other hand, the results of [11] observed that *Aspergillus* sp. had a high production ability for protease, followed by *Fusarium solani.* It was shown by [44] that *Mycelia sterilia* isolated from the roots of *Catharanthus rosues* was highly effective compared to other fungal isolates. In addition, the activity of protease was observed by [45], where it was shown that the activity was positive for 16 strains of *F. oxysporium* isolated from bananas. Interestingly, protease enzymes play an active role in the pathogenesis of fungi and fungal physiology to digest extracellular large peptides [46], as they work to degrade the proteins present in living tissues and help in penetration into the tissues of the host as well as breaking down glycoprotein in the cell wall. Protease enzymes are used in many medical applications for wounds, digestive health, blood clotting, diabetes, and other applications [47].

### 3.4. Cellulase Activity

**Figure 4:**
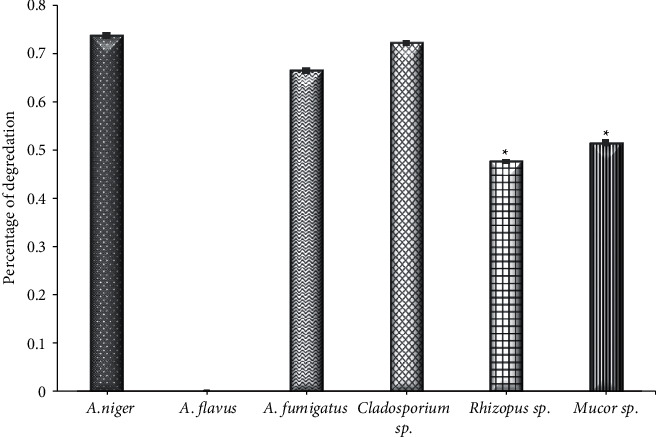
The activity of cellulase of endophytic fungi isolated from *Ziziphus spin*a leaves on solid medium. ^*∗*^Significant difference with (*A. Niger*, *A. flavus,* (A) *fumigatus, and Cladosparium sp.*), at *P* < 0.001.

Statistically, it was observed a significant difference (*P* < 0.001) between the cellulase-producing fungi. The results showed that *Rhizopus* sp. with a degradation percentage of 0.47325 and *Mucor* sp. (0.511) were able to produce cellulase enzymes, and the remaining strains exhibited a weak ability to produce this enzyme, while *A. flavus* could not produce the enzyme (Figure 4). The results of [37] found that out of 9 isolates, only 4 strains of endophytic fungi were able to produce cellulases, and one of them was *Aspergillus*. These results are in agreement with the results of [48], which found that some but not all endophytic fungi isolates are capable of producing cellulose. The results of the study [11] also showed that only 32% of the endophytic fungi were able to produce cellulose, which is consistent with the results of [49] among isolates from *Mangrove angiosporium*. The authors of [50] showed that 66% of the endophytic fungi isolated from *Brucea javanica* are capable of producing cellulose; [51] also showed that the activity of cellulases was 53.84% in endophytic fungi isolated from *Opunita ficus-indica*. It was indicated that the endophytic fungi have a good ability to secrete cellulases with pectinase, indicating that the fungus has a good ability to penetrate the cells of the live host as well as its ability to analyze dead components [11]. Continued demand for renewable, environmentally friendly fuel sources has intensified research into cellulose-degrading enzymes. The Earth's abundant cellulosic materials represent a promising and ideal energy source, so the extensive research and application of cellulolytic enzymes [52] makes them one of the most commercialized energy sources in the world [53].

### 3.5. Pectinase Activity

**Figure 5:**
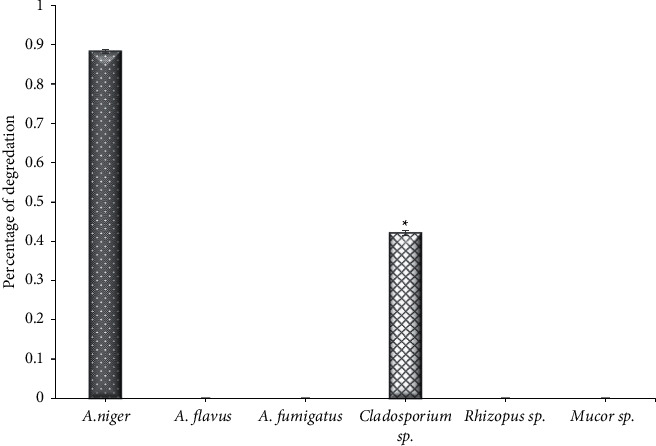
The activity of pectinase of endophytic fungi isolated from *Ziziphus spina* leaves on solid medium. ^*∗*^Significant difference with all other groups at *P* < 0.001.

The results of the current study showed a significant difference (*P* < 0.001) between the strains in terms of pectinase production. The results showed that the strain of *Cladosporium* sp. produced pectinase with a degradation percentage of 0.4213, while *A. niger* strain had a weak ability to produce this enzyme (0.883) and the remaining strains could not produce this enzyme (Figure 5). The results of [11] study on endophytic fungi in several medicinal plants showed that among the fungi that produced pectinase was *Taloromyces emersonii*, followed by *Fusarium oxysporium* in *Calophyllum inophyllum* plant. While the most productive fungi strains from *Alpinia calcarata* plant were *Fusicoccum* sp, *Myrothecium* sp*, Cylindrocephalum* sp., and *Colletotrichum gleosporoides* from *Bixa orellana* plant, the enzyme production was about 62% of the total fungi isolated from these plants. The pectinase enzyme is stimulated in both endophytic and pathogenic fungi by the availability or presence of the pectin substance. The pectinase is present in microorganisms, which is necessary for each of the pathogenic processes of plants, the symbiotic living between the organism and the plant, as well as in the processes of decomposition of dead plant materials [51]. The process of attacking the plant by pathogenic fungi begins through its ability to produce the pectinase, which plays a role in the decomposition of host tissues [54, 55]. The endophytic fungi can have the ability to transform into pathogenic fungi through their ability to decompose the pectin substances [50]. Additionally Schulz and Boyle [56] stated in their hypothesis that the interaction between the botanical host and the cultivated fungi takes place through a balance between the pathogenicity of the fungus and the defenses of the host plant, and the occurrence of any defect in this balanced is relative to the development of the disease or the occurrence of the disease. Pectinase also has important applications, especially in the paper and food industries, and is produced commercially through fungi [57].

### 3.6. Laccase Activity

The findings of this study showed that *A. niger* and *Cladosporium* sp. were the only fungi among the isolated fungi from *Ziziphus spina* leaves that showed a weak ability to produce laccase enzyme, while the other isolates did not show any ability to produce it. The results of [11] that found very few endophytic fungi can produce laccase, which is consistent with the results of both [30] and [57] studies [58], also showed that a small number of endophytic fungi can produce laccase enzyme. On the other hand, [59] observed that endophytic fungi isolates could not produce this enzyme, as for [1] which reported that there were only five strains out of 127 isolates that were isolated from eucalyptus trees that were positive in terms of their production of laccase. The nature of the endophytic fungi may be the reason for its loss of the ability to produce the laccase due to the activity of laccase which may harm the plant [11, 60]. The endophytic fungi that had the ability to produce laccase were fungi isolated from the root wood, such as *Trametes versicolor and T. villosa*, which are responsible for removing the toxic phenol from the medium in which these fungi grow under normal conditions [61]. Enzymes such as xylanases and laccases obtained from fungi have the ability to bio-bleach agriculture waste-based pulps by cleaving the *β*-1, 4 backbone of the complex plant cell wall for papermaking [62, 63].

## 4. Conclusion

The conclusion from this study is that the leaves of the *Ziziphus spina* medical plant contain different endophytic fungi that possess the ability to produce hydrolyze enzymes. The high activity of secreting some enzymes indicates the possibility of exploiting the enzymes of these fungi in many applications after their separation and characterization. The ability of endophytic fungi in the medicinal plant to produce extracellular enzymes shows high potential in therapeutic applications for clinical microbiology and can be used in biotechnological applications.

## Figures and Tables

**Table 1:** Endophytic fungi isolated from *Ziziphus spina* leaves and colonization frequency.

Endophytic fungi	Colony number	% colonization frequency (%C.F)	Chi-square (*ϰ*^2^)
*A. niger*	7	13.46	7
*A. fumigatus*	6	11.54	
*A. flavus*	3	5.77	
*Cladosporium* sp.	4	3.85	
*Rhizopus* sp.	2	7.69	
*Mucor* sp.	4	7.69	
Total number	**26**	**50%**	

**Table 2:** Enzymatic activity of endophytic fungi isolated from *Ziziphus spina* leaves on solid medium.

Endophytic fungi	Amylase	Protease	Cellulase	Lipase	Pectinase	Laccase
*A. niger*	**+++**	**+**	**+**	**++**	**+**	**+**
*A. flavus*	**+**	**+**	−	**+**	−	−
*A. fumigatus*	−	**+**	**+**	**+**	−	−
*Cladosporium* sp.	**+**	**++**	**+**	**++**	**++**	**+**
*Rhizopus* sp.	**++**	−	**++**	**+**	−	−
*Mucor* sp.	**++**	−	**++**	**+**	−	−
Percentage	**47.51%**	**44.75%**	**51%**	**68%**	**22%**	**25%**

Weak (^+^), moderate (^++^), high (^+++^), unable to produce(^−^).

## Data Availability

All data are presented within the article.
